# Increased stress hyperglycemia ratio at hospital admission in stroke patients are associated with increased in-hospital mortality and length of stay

**DOI:** 10.1186/s13098-024-01303-1

**Published:** 2024-03-16

**Authors:** Di Shen, Xintian Cai, Qing Zhu, Mulalibieke Heizhati, Junli Hu, Shuaiwei Song, Wenbo Yang, Jing Hong, Nanfang Li

**Affiliations:** 1 Hypertension Center of People’s Hospital of Xinjiang Uygur Autonomous Region, Xinjiang Hypertension Institute, Urumqi, Xinjiang 830001 People’s Republic of China; 2 NHC Key Laboratory of Hypertension Clinical Research, Urumqi, Xinjiang 830001 People’s Republic of China; 3 Key Laboratory of Xinjiang Uygur Autonomous Region “Hypertension Research Laboratory”, Urumqi, Xinjiang 830001 People’s Republic of China; 4 Xinjiang Clinical Medical Research Center for Hypertension (Cardio-Cerebrovascular) Diseases, Urumqi, Xinjiang 830001 People’s Republic of China

**Keywords:** Stroke, Stress hyperglycemia ratio, In-hospital mortality, Length of stay

## Abstract

**Objective:**

Recently, the stress hyperglycemia ratio (SHR) has been introduced as a metric to signify relative hyperglycemia. This study aimed to investigate the relationship between SHR and in-hospital mortality and length of stay occurring during hospitalization in stroke patients.

**Methods:**

The retrospective cohort study comprised a total of 4,018 patients diagnosed with acute stroke. The SHR is expressed by the formula: SHR = ABG (mmol/L) / [1.59 × HbA1c (%) − 2.59]. Outcomes included in-hospital mortality and length of stay. Multivariable logistic and linear regression analyses were conducted. Receiver operating characteristic (ROC) analysis was performed to distinguish between the variables, and the area under the ROC curve (AUC) was compared.

**Results:**

In this analysis, a total of 4,018 individuals participated, including 2,814 male patients, accounting for 70.0%. Overall, in-hospital mortality and length of stay tended to rise as SHR increased. A higher prevalence of in-hospital mortality was observed with each standard deviation (SD) increase of the SHR (odds ratio [OR]: 1.26, 95% confidence interval [CI]: 1.05–1.52). Moreover, after considering the confounders, a significant positive association between SHR levels and length of stay was observed (β = 0.70, 95% CI: 0.40-1.00). ROC analysis showed that among stroke patients, SHR (AUC = 0.693) was more effective than admission blood glucose (ABG) (AUC = 0.646) and glycosylated hemoglobin (HbA1c) (AUC = 0.523), which were more predictive of in-hospital mortality.

**Conclusions:**

Elevated SHR levels are associated with increased in-hospital mortality and prolonged length of stay in stroke patients.

**Supplementary Information:**

The online version contains supplementary material available at 10.1186/s13098-024-01303-1.

## Introduction

With high rates of disability and mortality, stroke is the primary common cause of adult disability and mortality in China [1, 2]. Given the current large demographic changes in China and the changing prevalence of vascular risk factors, it is expected that the mortality rate from stroke would rise sharply in the coming decades [3, 4]. The mortality rate of stroke patients in hospitals is high, and the length of stay is increasing. Therefore, it is crucial to carry out effective risk stratification for stroke patients in the hospital, establish optimal management strategies, and achieve accurate treatment [5, 6].

Stress hyperglycemia affects a significant proportion of stroke patients [7]. A rise in blood glucose levels has been noted in the acute phase of stroke. Stress hormones are released following activation of the sympathetic nervous system and the hypothalamus-pituitary-adrenal axis, causing this rise [8]. Previous research suggests that persistent hyperglycemia is associated with more adverse outcomes in stroke incidents [9]. Studies have shown that glycosylated hemoglobin (HbA1c) level was an independent predictor of worse functional outcome in patients with acute anterior circulation ischemic stroke (IS) [10, 11]. Persistent hyperglycemia has been linked to worse outcomes from strokes, according to prior study [9]. On the other hand, acute stress reactivity or persistent hyperglycemia may be the cause of increased glucose levels at the time of hospital admission [12]. To address this issue, a novel marker that more precisely reflects acute hyperglycemia has been created: the stress hyperglycemia ratio (SHR). The chronic glycemic value, which is determined using glycosylated hemoglobin (HbA1c), and the acute admission blood glucose (ABG) are the basis for estimating the SHR [13, 14]. Research indicates a significant independent correlation between SHR and adverse long-term outcomes in non-obstructive coronary artery patients [14]. Research has shed light on the influence of SHR on initial neurological impairments and the future outcomes for patients with acute IS [15]. Further research indicates that SHR serves as a reliable predictor of early hematoma growth and adverse outcomes in patients experiencing intracerebral hemorrhage [16]. SHR is suggested as a promising indicator for predicting unfavorable outcomes in critically ill patients by assessing the extent of stress-induced hyperglycemia in relation to the severity of illness [17]. However, the prognosis of patients with SHR and all types of stroke remains unclear. In addition, previous studies have limited attention given to the association between SHR and length of stay. Burdening society by prolonging hospitalization, which further increases the cost of healthcare [18]. However, no study has yet explored the possible connection between SHR and length of stay. Thus, we performed a retrospective analysis to look into the relationship between SHR and in-hospital mortality as well as the length of stay in patients with stroke.

## Materials and methods

### Study population

Between January 1, 2015, and December 31, 2022, 10,466 acute stroke patients in total were included in the research. The following were the study’s inclusion criteria: (1) age ≥ 18 years; (2) acute stroke [including acute IS and hemorrhagic stroke (HS)] verified by radiological examination [cranial computed tomography (CT) or magnetic resonance imaging (MRI)]. The following were the exclusion criteria: (1) incomplete data pertaining to age, sex, body mass index (BMI), and comorbidities; (2) patients with conditions that affect HbA1c levels, such as renal failure (serum creatinine concentration > 180 mmol/L) and anemia (hemoglobin < 100 g/L); (3) lack of baseline data for HbA1c or admission glucose levels, or measurements not taken within 24 h of admission; (4) ABG < 3.9 mmol/L; (5) patients who were discharged or died within 48 h of admission. Ultimately, 4018 participants were included in the analysis (Figure S1). All patients (or their legal relatives) who participated in the study gave written informed permission, and the study was approved by the hospital’s ethics committee. We followed the STROBE statement in reporting this study [19].

### Data collection and definition

Baseline data, including clinical information, lifestyle variables, test findings, medical history, and medication history, were taken from the electronic medical record. Clinical data included age, sex, height, weight, BMI, systolic, diastolic, and heart rate at admission. Both smoking and alcohol drinking were classified as current or non-current.

Laboratory indicators include white blood cells (WBC), red blood cells (RBC), hemoglobin (HB), platelets (PLT), alanine aminotransferase (ALT), aspartate aminotransferase (AST), albumin/globulin ratio (A/G), serum creatinine (Scr), uric acid (UA), serum potassium, serum sodium, total cholesterol (TC), triglycerides (TG), high-density lipoprotein cholesterol (HDL-C), low-density lipoprotein cholesterol (LDL-C), and HbA1c. The ABG was defined as the first available plasma glucose measurement within a day after admission. Relative hyperglycemia was determined by the SHR, calculated as the admission blood glucose (ABG) divided by the estimated average glucose [13]. The SHR is expressed by the formula: SHR = ABG (mmol/L) / [1.59 × HbA1c (%) − 2.59] [13]. The above blood biochemical indicators were detected by Automatic Analyzer (7600-010, Hitachi, Tokyo, Japan) according to the manufactures instruction.

The definition of hypertension was a self-reported history of medical diagnosis and antihypertensive drug usage. Dyslipidemia was defined by self-report dyslipidemia and/or treatment with a lipid-lowering drug. In case the participant disclosed a history of diabetes mellitus or used anti-hyperglycemic medication, diabetes mellitus was deemed to be present. The diagnosis of coronary artery disease was based on the patient’s self-reported history of myocardial infarction, percutaneous coronary intervention, or coronary bypass grafting. An ischemic or hemorrhagic stroke history was considered a prior stroke.

### Outcomes

The outcome measures for this study included the length of stay and in-hospital mortality. Whatever the cause of death, every death that happened while the patient was in the hospital was considered to be an instance of in-hospital mortality. The time from admission to discharge was considered the length of stay. The medical records of stroke patients were identified by trained staff using the International Classification of Diseases, Tenth Revision (ICD-10) codes. The diagnosis and classification of acute stroke were determined based on WHO criteria, in combination with confirmation through brain CT or MRI [20, 21].

### Statistical analysis

The χ^2^ test was used for categorical variables, and for continuous variables, either ANOVA or the Kruskal-Wallis test was employed.

We calculated the variance inflation factor (VIF) for all covariates (Table S1). Continuous outcomes were analyzed using multivariable linear regression, and binary outcomes were analyzed using multivariable logistic regression. Tests for trend were calculated using the tertile number as a continuous variable. Spearman’s correlations were employed to assess the association between the risk factors linked to in-hospital mortality and length of stay and SHR levels as a continuous variable. Restricted cubic spline (RCS) models were fitted for both linear and logistic regression models. To assess potential interactions, stratified analyses were carried out. Sensitivity analyses were employed to test the robustness of the results. Furthermore, the receiver operating characteristics (ROC) curve was plotted to evaluate the predictive utility of SHR for in-hospital mortality compared to ABG and HbA1c. Statistical analysis was performed using R 4.1.1 software. We considered *p* < 0.05 (two sided) as significant.

## Results

### Baseline characteristics

**Fig. 1:**
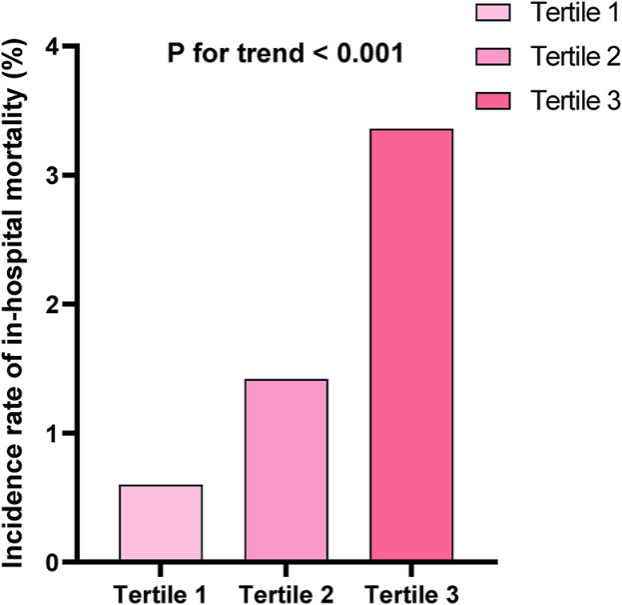
Incidence of in-hospital mortality according to the tertiles of SHR

**Fig. 2:**
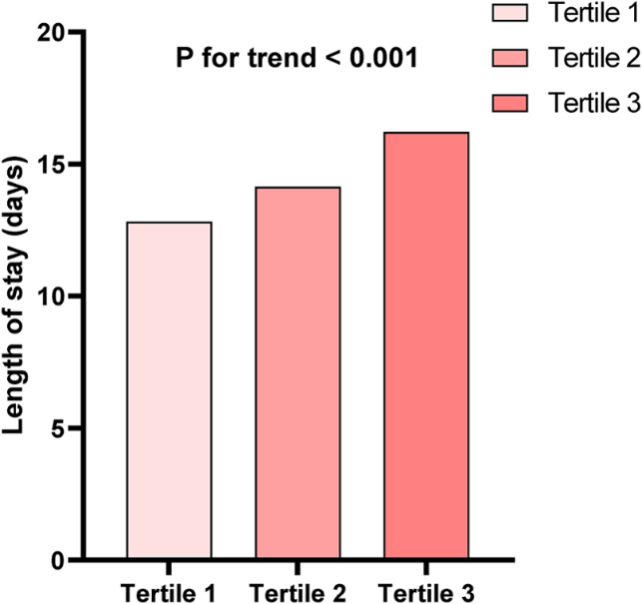
Length of stay according to the tertiles of SHR

The baseline parameters for each group, according to the tertiles of SHR, are presented in Table 1. A total of 4018 individuals participated in this analysis, with 2814 male patients (70.0%) included. The incidence of in-hospital mortality and length of stay tended to increase with increasing SHR (Figs. 1 and 2). Overall, the distribution of SHR in the population was normal (Figure S2). Higher levels of SHR were linked to age, sex, current smoking, current drinking, higher BMI, hypertension, and diabetes mellitus. The group with elevated SHR exhibited significantly increased heart rate, SBP, and DBP. In terms of laboratory parameters, WBC, HB, PLT, UA, serum potassium, TC, TG, HDL-C, LDL-C, HbA1c, and ABG increased with SHR (Table 1). To investigate the risk factors associated with the relationship between SHR levels and in-hospital all-cause death and length of stay, we conducted Pearson correlation analysis (Table S2). In this analysis, SBP, DBP, WBC, serum creatinine, TC, TG, HDL-C, HbA1c, and ABG were found to be positively correlated with SHR levels. ABG exhibited the strongest association (*r* = 0.739).

**Table 1:** Baseline characteristics according to tertiles of SHR

Variables	T1	T2	T3	P-value
	(0.74<)	(0.74–0.93)	(> 0.93)	
Sample size, n	1339	1339	1340	
**Demography**				
Age, years	57.47 ± 7.93	56.43 ± 8.21	57.29 ± 8.09	0.002
Sex				0.013
Women	423 (31.59%)	361 (26.96%)	420 (31.34%)	
Men	916 (68.41%)	978 (73.04%)	920 (68.66%)	
Current smoking	451 (33.68%)	492 (36.74%)	397 (29.63%)	< 0.001
Current drinking	313 (23.38%)	335 (25.02%)	278 (20.75%)	0.030
Heart rate, beats/min	79.84 ± 12.76	79.77 ± 13.00	82.61 ± 14.33	< 0.001
BMI, kg/m^2^	26.49 ± 3.69	26.65 ± 3.61	26.18 ± 3.65	0.003
SBP, mmHg	141.48 ± 22.36	145.16 ± 23.43	149.49 ± 25.38	< 0.001
DBP, mmHg	84.81 ± 13.82	87.23 ± 14.52	88.45 ± 15.13	< 0.001
**Previous history**				
Hypertension	550 (41.08%)	535 (39.96%)	622 (46.42%)	0.001
Coronary artery disease	111 (8.29%)	87 (6.50%)	106 (7.91%)	0.181
Diabetes mellitus	265 (19.79%)	206 (15.38%)	361 (26.94%)	< 0.001
Previous haemorrhagic stroke	37 (2.76%)	34 (2.54%)	39 (2.91%)	0.839
Previous ischemic stroke	127 (9.48%)	99 (7.39%)	116 (8.66%)	0.149
**Biochemical indexes**				
WBC, 10^9/L	7.51 ± 2.23	7.60 ± 2.34	8.36 ± 2.66	< 0.001
RBC, 10^12/L	4.77 ± 0.53	4.80 ± 0.51	4.76 ± 0.57	0.121
HB, g/L	143.61 ± 17.43	146.18 ± 17.02	146.43 ± 18.74	< 0.001
PLT, 10^9/L	246.81 ± 63.38	236.15 ± 61.75	235.12 ± 64.35	< 0.001
ALT, U/L	22.00 (16.00–33.00)	23.00 (16.50–33.00)	23.00 (16.00–33.00)	0.411
AST, U/L	19.00 (16.00-24.90)	20.00 (16.00-24.91)	20.00 (16.00–26.00)	0.194
Albumin/globulin ratio	1.44 ± 0.27	1.48 ± 0.27	1.42 ± 0.27	< 0.001
Serum creatinine, µmol/L	70.28 ± 27.02	71.51 ± 26.78	72.74 ± 30.74	0.078
Uric acid	319.68 ± 90.81	331.72 ± 92.18	321.81 ± 98.98	0.002
Serum potassium, mEq/L	3.85 ± 0.39	3.86 ± 0.41	3.88 ± 0.41	0.076
Serum sodium, mEq/L	140.33 ± 3.27	140.28 ± 3.11	139.30 ± 4.09	< 0.001
Total cholesterol, mmol/L	4.05 ± 1.07	4.23 ± 1.10	4.37 ± 1.14	< 0.001
Triglyceride, mmol/L	1.39 (1.04–1.92)	1.52 (1.10–2.13)	1.62 (1.13–2.45)	< 0.001
HDL-C, mmol/L	0.96 ± 0.25	0.99 ± 0.26	1.01 ± 0.30	< 0.001
LDL-C, mmol/L	2.40 ± 0.90	2.48 ± 0.91	2.52 ± 0.94	0.003
HbA1c, %	6.89 ± 1.71	6.57 ± 1.63	7.21 ± 2.13	< 0.001
Admission blood glucose, mmol/L	5.19 ± 1.57	6.47 ± 2.23	10.94 ± 5.70	< 0.001
Type of stroke, n (%)				0.007
Ischemic stroke	1031 (77.00%)	1043 (77.89%)	1018 (75.97%)	
Haemorrhagic stroke	308 (23.00%)	296 (22.11%)	322 (24.03%)	

*Notes* Data are mean (standard deviation), n (%), or median (interquartile range)

*Abbreviations* BMI, body mass index; SBP, systolic blood pressure; DBP, diastolic blood pressure; WBC, white blood cells; RBC, red blood cells; HB, hemoglobin; PLT, platelets; ALT, alanine aminotransferase; AST, aspartate aminotransferase; HDL-C, high-density lipoprotein cholesterol; LDL-C, low-density lipoprotein cholesterol; HbA1c, glycosylated hemoglobin

### Relationship between SHR and in-hospital mortality

**Fig. 3:**
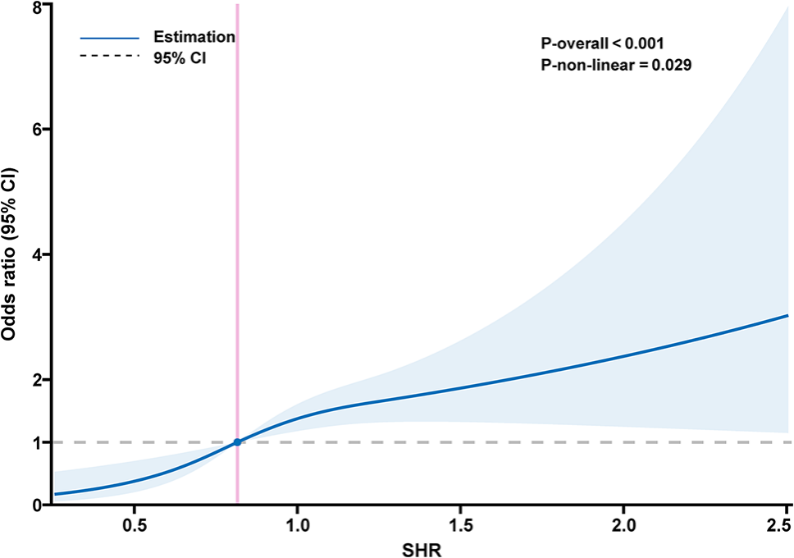
Restricted cubic spline for the association between SHR and in-hospital mortality in patients with acute stroke

Overall, we found that among stroke patients, elevated SHR was substantially and positively associated with in-hospital mortality. With an odds ratio (OR) of 1.53 (95% CI: 1.32–1.77, *p* < 0.001) in the crude model, SHR levels were substantially linked with in-hospital mortality. After adjusting for confounding factors in the Model 3, SHR remained an independent risk factor for in-hospital mortality, with an OR of 1.26 (95% CI: 1.05–1.52, *p* < 0.001) (Table 2). In both acute IS and acute HS, similar outcomes were seen (Tables S3-S4). After excluding individuals with missing covariates, the association was not materially changed (Table S5). Furthermore, to evaluate the sensitivity to unmeasured confounding, we produced E-values (Table S6 and Figure 3). We further found a significant dose–response relationship between SHR and in-hospital mortality (*p* for nonlinear association = 0.029) (Fig. 3).

**Table 2:** Associations of SHR and in-hospital mortality in patients with acute stroke

Exposure	Crude model	Model 1	Model 2	Model 3
	Odds ratio (95% CI)	Odds ratio (95% CI)	Odds ratio (95% CI)	Odds ratio (95% CI)
Continuous (per 1-SD increase)	**1.53 (1.32, 1.77)**	**1.43 (1.23, 1.68)**	**1.31 (1.10, 1.55)**	**1.26 (1.05, 1.52)**
SHR median				
≤ 0.81	Reference	Reference	Reference	Reference
> 0.81	**3.57 (2.04, 6.25)**	**3.12 (1.77, 5.51)**	**2.72 (1.51, 4.90)**	**2.48 (1.37, 4.50)**
SHR tertiles				
T1 (0.74<)	Reference	Reference	Reference	Reference
T2 (0.74–0.93)	**2.39 (1.04, 5.49)**	**2.39 (1.04, 5.50)**	**2.40 (1.03, 5.58)**	2.28 (0.98, 5.33)
T3 (> 0.93)	**5.78 (2.72, 12.31)**	**5.00 (2.33, 10.73)**	**4.34 (1.98, 9.52)**	**3.89 (1.76, 8.62)**
P for trend	< 0.001	< 0.001	< 0.001	< 0.001

Model 1: adjusted for age, sex, smoking status, alcohol consumption, heart rate, BMI, SBP, and DBP

Model 2: adjusted for variables in Model 1 + WBC, RBC, HB, PLT, triglyceride, HDL-C, LDL-C, HbA1c, ALT, AST, GGT, albumin/globulin ratio, Scr, BUN, UA, potassium, and sodium

Model 3: adjusted for variables in Model 2 + hypertension, coronary artery disease, diabetes mellitus, cancer, previous haemorrhagic stroke, and previous ischemic stroke

*Abbreviations* SD = standard deviation. Other abbreviations, see Table 1

### Relationship between SHR and length of stay

**Fig. 4:**
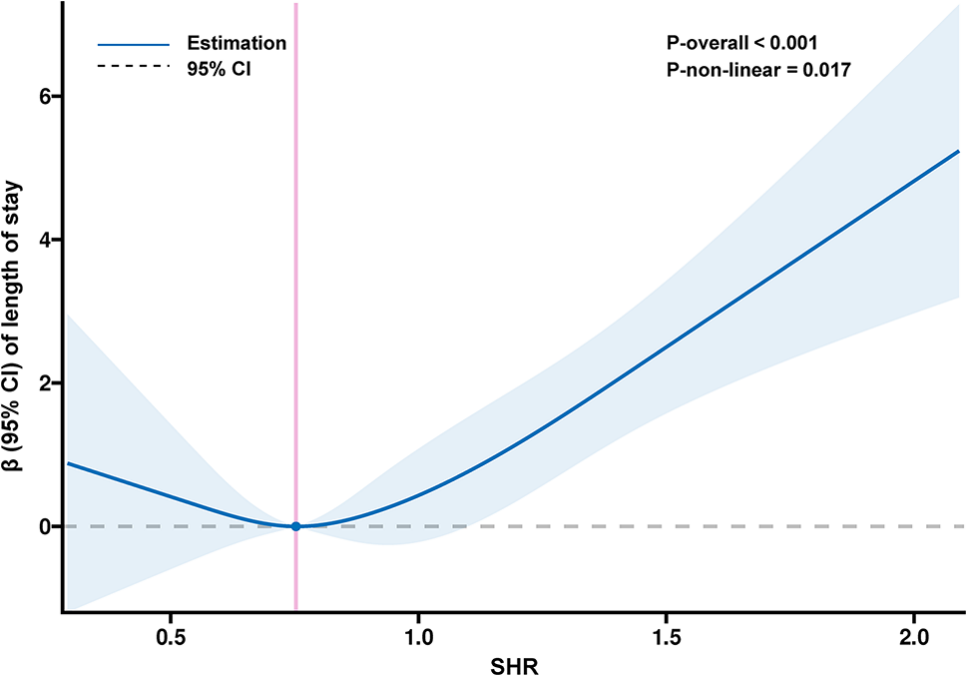
Restricted cubic spline for the association between SHR and length of hospital stay in patients with acute stroke

Univariate and multivariable-adjusted linear regression models were applied to assess the association between SHR levels and length of stay. In the crude model, SHR levels were substantially correlated with length of stay (β = 0.83, 95% CI: 0.55–1.12, *p* < 0.001). The fully adjusted model (β = 0.70, 95% CI: 0.40–1.00, *p* = 0.022) did not change the results (Table 3). Comparable outcomes were found in acute IS and HS (Tables S7-S8). After excluding participants with missing covariates, the results did not significantly alter (Table S9 and Figure S4). In addition, the main results were robust, and we produced E-values to evaluate the sensitivity to unmeasured confounding (Table S10 and Figure S4). After adjustment for multiple covariates, we found a significant dose-response relationship between SHR and length of stay (*p* for non-linear association *p* = 0.017) (Fig. 4).

**Table 3:** Associations of SHR and length of hospital stay in patients with acute stroke

Exposure	Crude model	Model 1	Model 2	Model 3
	β (95% CI)	β (95% CI)	β (95% CI)	β (95% CI)
Continuous (per 1-SD increase)	**0.83 (0.55, 1.12)**	**0.77 (0.48, 1.06)**	**0.72 (0.43, 1.01)**	**0.70 (0.40, 1.00)**
SHR median				
≤ 0.81	Reference	Reference	Reference	Reference
> 0.81	**1.35 (0.77, 1.92)**	**1.20 (0.62, 1.78)**	**1.08 (0.50, 1.65)**	**0.92 (0.33, 1.50)**
SHR tertiles				
T1 (0.74<)	Reference	Reference	Reference	Reference
T2 (0.74–0.93)	0.32 (-0.38, 1.03)	0.15 (-0.55, 0.86)	0.20 (-0.51, 0.90)	0.37 (-0.32, 1.06)
T3 (> 0.93)	**1.40 (0.70, 2.11)**	**1.19 (0.48, 1.91)**	**1.08 (0.37, 1.79)**	**0.84 (0.12, 1.56)**
P for trend	< 0.001	0.001	0.003	0.022

Model 1: adjusted for age, sex, smoking status, alcohol consumption, heart rate, BMI, and DBP

Model 2: adjusted for variables in Model 1 + WBC, RBC, HB, PLT, triglyceride, HDL-C, LDL-C, HbA1c, ALT, AST, GGT, albumin/globulin ratio, Scr, BUN, UA, potassium, and sodium

Model 3: adjusted for variables in Model 2 + hypertension, coronary artery disease, diabetes mellitus, cancer, previous haemorrhagic stroke, and previous ischemic stroke

*Abbreviations* SD = standard deviation. Other abbreviations, see Table 1

### Subgroup analysis

**Fig. 5:**
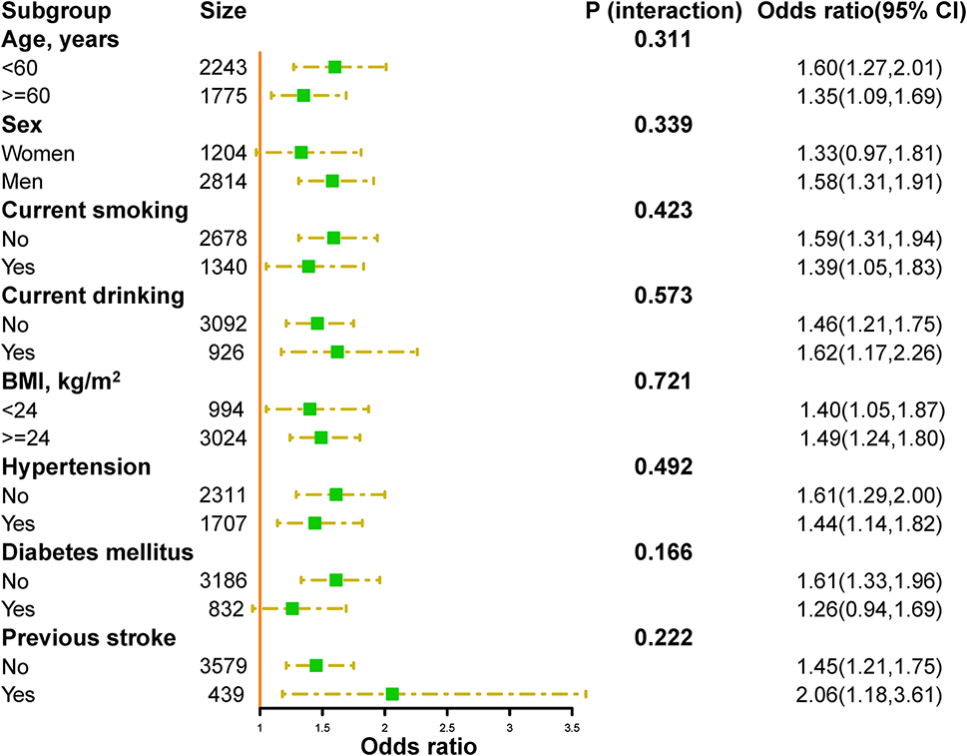
Stratified analyses of the association between SHR (per SD increment) and in-hospital mortality in patients with acute stroke

**Fig. 6:**
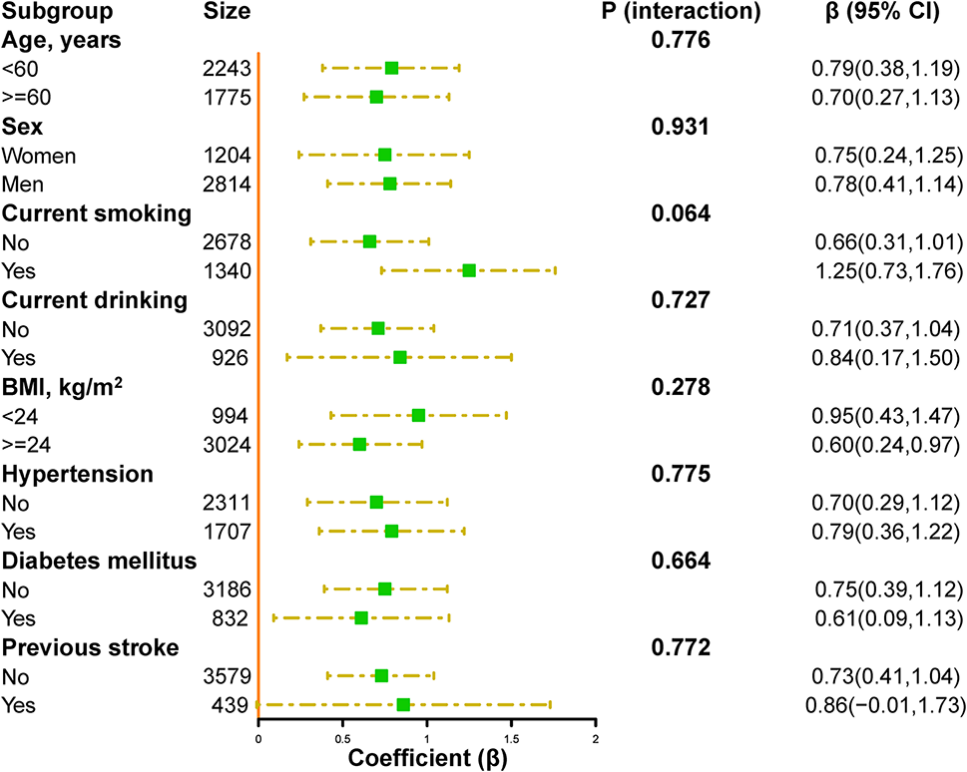
Stratified analyses of the association between SHR (per SD increment) and length of hospital stay in patients with acute stroke

To investigate the potential interactions between SHR and related factors on in-hospital mortality, further stratified analyses were conducted (Fig. 5). None of the variables, including age, sex, current smoking, current drinking, BMI, history of hypertension, history of diabetes mellitus, and history of prior stroke, significantly modified the relationship between SHR and in-hospital mortality (all *p* for interactions > 0.05). We performed further stratified analyses to assess the relationship between SHR and length of stay in various subgroups (Fig. 6). However, no statistically significant interaction was observed.

### ROC analysis

**Fig. 7:**
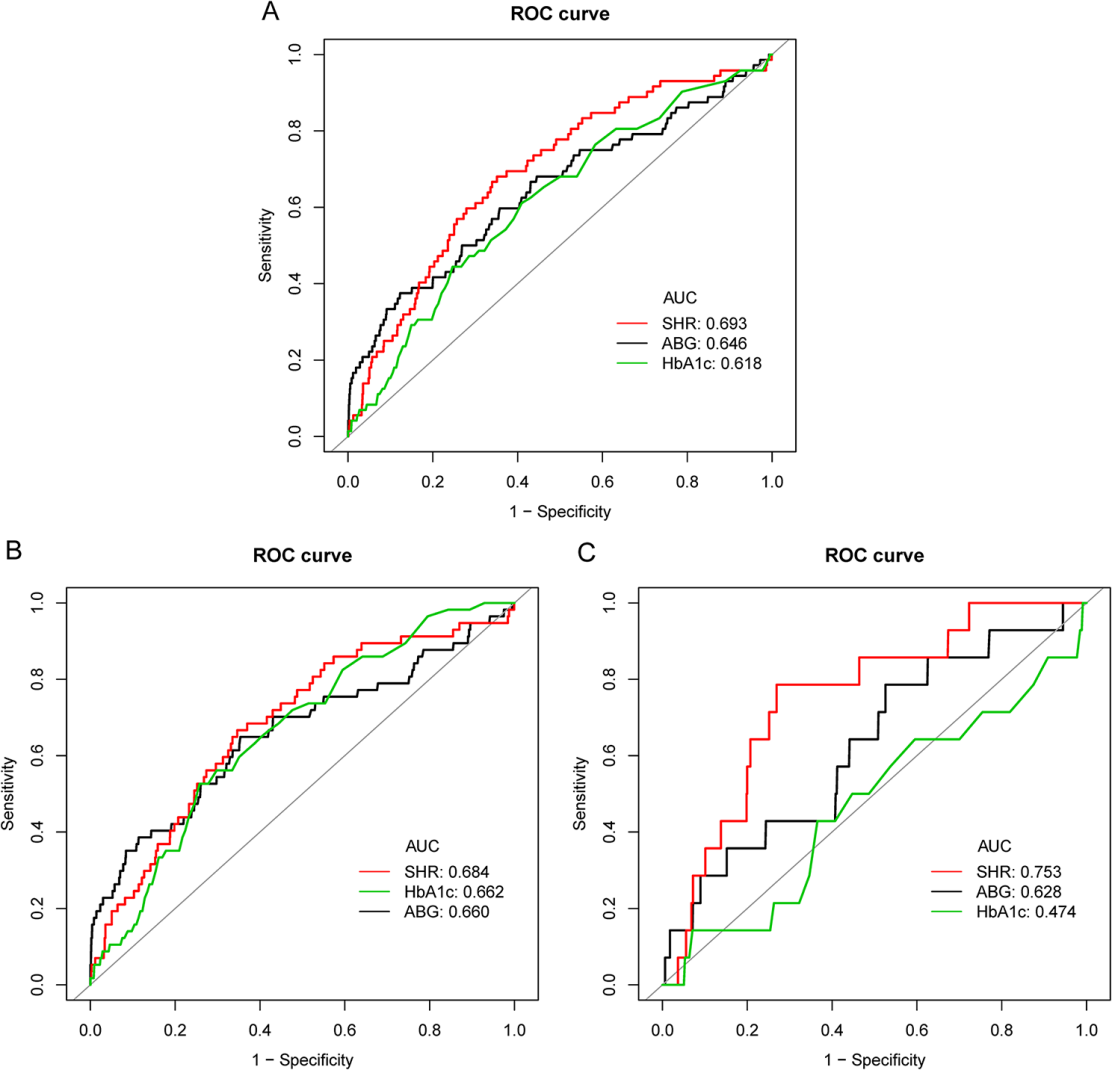
Receiver operating characteristic (ROC) analysis of SHR, admission glucose, and HbA1c for predicting in-hospital mortality. **A**: In patients with acute stroke; **B**: In patients with acute ischemic stroke; **C**: In patients with acute haemorrhagic stroke

According to ROC analysis, SHR [area under the curve (AUC) = 0.693] was a stronger predictor of in-hospital mortality in all stroke patients than ABG (AUC = 0.646) and HbA1c (AUC = 0.523) (Fig. 7A). Comparable outcomes were obtained in patients with acute IS (Fig. 7B) and HS (Fig. 7C).

## Discussion

This is the first study to examine the relationship between SHR and in-hospital mortality and length of stay in stroke patients. The current study has two major findings. First, among stroke patients, those with high SHR had higher in-hospital mortality and length of stay. Second, SHR is independently associated with in-hospital mortality and length of stay in ischemic and hemorrhagic stroke patients.

The study determined that the SHR is a more effective quantitative indicator for stress hyperglycemia than blood glucose levels when assessing critical illness outcomes^11^. Previous research has demonstrated a negative correlation between stress hyperglycemia and poor prognosis in patients with acute myocardial infarction [22–24]. P. A. O’Neill et al. have previously determined the stress hormone and blood glucose response after acute stroke and found that irritability and blood glucose increases in non-diabetic patients are signs of a poor prognosis after stroke [25]. A recent meta-analysis demonstrated that higher SHR levels significantly increase the rates of poor prognosis, mortality, neurologic deficits, hypertension, and infectious complications, regardless of diabetes or whether patients underwent intravenous thrombolysis or mechanical thrombectomy [26]. In a prospective study, Konstantinos Tziomalos et al. discovered that stress hyperglycemia is linked to more severe acute IS and that such patients had more adverse functional outcomes and higher in-hospital mortality at discharge^13^. These findings suggest that SHR could be a significant factor in patient prioritizing and a valid biomarker for predicting in-hospital mortality in stroke populations. Acute hyperglycemia following a stroke might be a risk factor for mortality since poor glycemic control seems to be linked to post-stroke dysfunction. Cao et al. found that hyperglycemia can enhance brain damage through multiple mechanisms, prolong length of hospital stay, increase recurrence and mortality rates, and negatively impact prognosis [27]. Other studies have also shown that the longer critically ill patients stay in the emergency department, the higher their morbidity and mortality rates [28]. Therefore, early identification of novel risk factors during the early stages of cerebral infarction and the implementation of hypoglycemic treatments are critical to reducing the harmful consequences of hyperglycemia.

Several potential mechanisms may account for the observed associations. First, stress hyperglycemia might be involved in the production of molecules that are inflammatory and vasoconstrictive, aggravating the oxidative stress that follows, causing mitochondrial malfunction, and results in endothelial dysfunction [29–31]. Second, hyperglycemia also activates protein kinase C and NADPH oxidase, increasing reactive oxygen species (ROS) levels and reducing nitric oxide synthase, which cause decreased reperfusion and aggravate more neurons to be injured [32]. Third, patients with acute IS often suffer from lipid metabolism disorders. Under the high blood sugar situation, macrophages swallow the glycated LDL, which will be transformed into foam cells, adhering to the blood vessel wall, thus contributing to the overlapping interaction of lipotoxicity, glucotoxicity, and inflammation, and causing poor clinical outcomes [33]. Finally, hyperglycemia may be directly neurotoxic to the ischemic penumbra, causing more neurons to be damaged. This might then cause hypoperfused at-risk tissue to infarct, thus resulting in poorer stroke outcome [34–36]. This would also indicate that SHR was a target for treatment in an attempt to alleviate the dismal prognosis associated with stroke.

### Strengths and limitations

Our study has several strengths, including its large sample size, consistent data collection methods, and comprehensive information on potential confounders. The study, however, is not without limitations. First, as this is a retrospective, single-center investigation, further patient cohorts need be used to confirm our findings. Second, a direct causal relationship cannot be inferred due to the study’s design. Third, the study failed to gather data on the hypoglycemic treatment administered to the participants, thereby preventing the assessment of any additional correlations between SHR and the hypoglycemic treatment. Fourth, since SHR was only measured at one moment in time, we were unable to account for variations in SHR over time, which could possibly affect in-hospital mortality. Fifth, we did not collect data on enteral nutrition, corticosteroids, or other interventions that might affect in-hospital mortality. Sixth, due to the absence of baseline scores from the National Institutes of Health Stroke Scale (NIHSS) and the Glasgow Coma Scale (GCS) in our dataset, our analysis could not ascertain the influence of SHR on NIHSS and GCS scores.

## Conclusion

This study demonstrated that SHR was associated with increased in-hospital mortality and prolonged length of stay in stroke patients. Thus, more research is necessary to validate our results.

### Electronic supplementary material

Below is the link to the electronic supplementary material.

Supplementary Material 1

## Data Availability

The datasets used and/or analyzed in the current study are available from the corresponding author upon reasonable request.

## Abbreviations

SHR: Stress hyperglycemia ratio
ROC: Receiver operating characteristic
AUC: Area under the ROC curve
SD: Standard deviation
OR: Odds ratio
CI: Confidence interval
HbA1c: Glycosylated hemoglobin
ABG: Admission blood glucose
IS: Ischemic stroke
HS: Hemorrhagic stroke
CT: Cranial computed tomography
MRI: Magnetic resonance imaging
BMI: Body mass index
WBC: White blood cells
HB: Hemoglobin
RBC: Red blood cells
PLT: Platelets
ALT: Alanine aminotransferase
AST: Aspartate aminotransferase
A/G: Albumin/globulin ratio
Scr: Serum creatinine
UA: Uric acid
TC: Total cholesterol
TG: Triglycerides
HDL-C: High-density lipoprotein cholestero
LDL-C: Low-density lipoprotein cholesterol
ICD-10: International Classification of Diseases, Tenth Revision
VIF: Variance inflation factor
RCS: Restricted cubic spline
