# Supplementary material for: Increased stress hyperglycemia ratio at hospital admission in stroke patients are associated with increased in-hospital mortality and length of stay
Source: Diabetol Metab Syndr. 2024 Mar 16;16:69. doi: 10.1186/s13098-024-01303-1 (PMC10943784; doi:10.1186/s13098-024-01303-1)
Supplement: Supplementary file 1 — Supplementary Material 1 [file 13098_2024_1303_MOESM1_ESM.docx]

Supplementary Material

# Supplemental material and methods

**Details of the statistical analyses**.

Clinical and demographic characteristics were reported using proportions, the mean and standard deviation (SD), or the median and interquartile range (IQR), as appropriate. To compare the characteristics among different SHR groups, the chi-square test was performed for categorical variables, and one-way analysis of variance, or the Kruskal-Wallis test, was performed for continuous variables with normal and skewed distributions. We used nonparametric missing value imputation, based on the missForest procedure in R, to account for missing data. A random forest model using the rest of the variables in the data set was performed to predict the missing values for these variables. The internally cross-validated errors were also estimated.

Multicollinearity in the resulting models was quantified using the variance inflation factor (VIF)—variables with VIF ≥ 5 were removed (Table S1). Spearman's correlations were employed to assess the association between the risk factors linked to in-hospital mortality and length of stay and SHR levels as a continuous variable. Then multivariable logistic regression models [odds ratio (OR) and 95% confidence interval (CI)] were used to evaluate the relationship between SHR and in-hospital mortality. Regression coefficients (β) and 95% CI were calculated from multivariable linear regression models to analyze the association between SHR and length of hospital stay. Crude model of the two regression models did not adjust for covariates; model 1 adjusted for age, sex, smoking status, alcohol consumption, heart rate, BMI, SBP, and DBP; model 2 adjusted for variables in Model 1+ WBC, RBC, HB, PLT, triglyceride, HDL-C, LDL-C, HbA1c, ALT, AST, GGT, albumin/globulin ratio, Scr, BUN, UA, potassium, and sodium; and model 3 adjusted for variables in Model 2+ hypertension, coronary artery disease, diabetes mellitus, cancer, previous haemorrhagic stroke, and previous ischemic stroke. P value for trend was calculated by treating tertiles as a continuous variable in each model. We used restricted cubic spline (RCS) models fitted for linear regression models and logistic regression models. Knots were present at the 5th, 25th, 75th, and 95th percentiles; the reference SHR of the logistic regression model was the median. The RCS, based on the linear regression model, was used to model the association between SHR and length of hospital stay.

As additional exploratory analyses, possible modifications of the association between SHR and in-hospital mortality and length of hospital stay were also assessed for variables including sex, age (<60 compared with ≥60 y), BMI (in kg/m^2^; <24 compared with ≥24), current smoking (yes or no), current drinking (yes or no), hypertension (yes or no), diabetes mellitus (yes or no), and previous stroke (yes or no). The statistical significance of an interaction term was evaluated by using the likelihood ratio test. We did two sensitivity analyses. First, to assess the impact of missing data, we repeated the preliminary analysis after excluding subjects with missing covariates at baseline. Second, we calculated E-values to evaluate robustness of the results to potential unmeasured confounding ^[1]^. Furthermore, the receiver operating characteristics (ROC) curve was plotted to examine the predictive utility of SHR for in-hospital mortality compared to admission glucose and HbA1c.

The statistical analysis was performed using software R, version 4.1.1 (R Foundation for Statistical Computing), and significance was set at a 2-sided P value less than .05.

# Supplementary Figures and Tables

## Supplementary Tables

**Table S1.** Collinearity diagnostics steps.

|  | Step 1 | Step 2 |
| --- | --- | --- |
| SHR | 9.1 | 1.2 |
| Age | 1.4 | 1.4 |
| Sex | 1.9 | 1.9 |
| Current smoking | 1.9 | 1.9 |
| Current drinking | 1.7 | 1.7 |
| Heart rate | 1.2 | 1.2 |
| BMI | 1.1 | 1.1 |
| SBP | 2.3 | 2.3 |
| DBP | 2.5 | 2.5 |
| Hypertension | 1.4 | 1.4 |
| Coronary artery disease | 1.1 | 1.1 |
| Diabetes mellitus | 1.6 | 1.6 |
| Previous haemorrhagic stroke | 1.0 | 1.0 |
| Previous ischemic stroke | 1.1 | 1.1 |
| WBC | 1.3 | 1.3 |
| RBC | 3.6 | 3.6 |
| HB | 4.4 | 4.4 |
| PLT | 1.4 | 1.4 |
| ALT | 3.3 | 3.3 |
| AST | 3.4 | 3.3 |
| Albumin/globulin ratio | 1.8 | 1.8 |
| Serum creatinine | 2.3 | 2.3 |
| Uric acid | 1.5 | 1.5 |
| Serum potassium | 1.2 | 1.2 |
| Serum sodium | 1.9 | 1.9 |
| Total cholesterol | **10.4** | **NA** |
| Triglyceride | 3.0 | 3.0 |
| HDL-C | 2.1 | 2.1 |
| LDL-C | **6.9** | **NA** |
| HbA1c | **8.8** | **NA** |
| Admission blood glucose | **17.6** | **NA** |

VIF = 1/(1-R^2^). VIF step-by-step screening method: Calculate the VIF of each variable. If the maximum VIF value ≥ 5, remove the variable with the maximum VIF value.

Abbreviations: VIF, variance inflation factor. Other abbreviations as presented in Table 1.

**Table S2.** Spearman's correlation between SHR levels and the risk factors associated with in-hospital mortality and length of stay in all subjects.

| Variables | Correlation coefficient (95% CI) | P-value |
| --- | --- | --- |
| Age | 0.002 (-0.03, 0.03) | 0.860 |
| BMI | -0.062 (-0.09, -0.03) | 0.000 |
| SBP | 0.119 (0.09, 0.15) | <0.001 |
| DBP | 0.071 (0.04, 0.10) | <0.001 |
| WBC | 0.142 (0.11, 0.17) | <0.001 |
| RBC | -0.048 (-0.08, -0.02) | 0.002 |
| HB | 0.021 (-0.01, 0.05) | 0.181 |
| PLT | -0.078 (-0.11, -0.05) | <0.001 |
| ALT | 0.005 (-0.03, 0.04) | 0.751 |
| AST | 0.045 (0.01, 0.04) | 0.004 |
| Albumin/globulin ratio | -0.056 (-0.09, -0.03) | 0.002 |
| Serum creatinine | 0.076 (0.05, 0.11) | <0.001 |
| Uric acid | 0.024 (-0.01, 0.06) | 0.126 |
| Serum potassium | 0.058 (0.03, 0.09) | 0.000 |
| Serum sodium | -0.134 (-0.16, -0.10) | <0.001 |
| Total cholesterol | 0.120 (0.08, 0.15) | <0.001 |
| Triglyceride | 0.144 (0.11, 0.17) | <0.001 |
| HDL-C | 0.088 (0.06, 0.12) | <0.001 |
| LDL-C | 0.040 (0.01, 0.07) | 0.012 |
| HbA1c | 0.111 (0.08, 0.14) | <0.001 |
| Admission blood glucose | 0.739 (0.72, 0.75) | <0.001 |

Abbreviations: Abbreviations as presented in Table 1.

**Table S3.** Associations of SHR and in-hospital mortality in patients with acute ischemic stroke.

| Exposure | Crude model | Model 1 | Model 2 | Model 3 |
| --- | --- | --- | --- | --- |
|  | Odds ratio (95% CI) | Odds ratio (95% CI) | Odds ratio (95% CI) | Odds ratio (95% CI) |
| Continuous (per 1-SD increase) | 1.58 (1.32, 1.89) | 1.47 (1.22, 1.77) | 1.34 (1.10, 1.64) | 1.32 (1.07, 1.64) |
| SHR median |  |  |  |  |
| ≤0.81 | Reference | Reference | Reference | Reference |
| >0.81 | 3.50 (1.88, 6.52) | 3.18 (1.69, 5.98) | 2.73 (1.41, 5.30) | 2.65 (1.34, 5.22) |
| SHR tertiles |  |  |  |  |
| T1 (0.74<) | Reference | Reference | Reference | Reference |
| T2 (0.74-0.93) | 2.83 (1.11, 7.21) | 3.02 (1.18, 7.72) | 3.37 (1.29, 8.84) | 3.26 (1.22, 8.70) |
| T3 (>0.93) | 5.90 (2.47, 14.12) | 5.37 (2.22, 12.97) | 4.74 (1.90, 11.83) | 4.66 (1.82, 11.91) |
| P for trend | <0.001 | <0.001 | 0.001 | 0.001 |

Model 1: adjusted for age, sex, smoking status, alcohol consumption, heart rate, BMI, SBP, and DBP.

Model 2: adjusted for variables in Model 1+ WBC, RBC, HB, PLT, triglyceride, HDL-C, LDL-C, HbA1c, ALT, AST, GGT, albumin/globulin ratio, Scr, BUN, UA, potassium, and sodium.

Model 3: adjusted for variables in Model 2+ hypertension, coronary artery disease, diabetes mellitus, cancer, previous haemorrhagic stroke, and previous ischemic stroke.

Abbreviations: SD = standard deviation. Other abbreviations, see Table 1.

**Table S4.** Associations of SHR and in-hospital mortality in patients with acute haemorrhagic stroke.

| Exposure | Crude model | Model 1 | Model 2 | Model 3 |
| --- | --- | --- | --- | --- |
|  | Odds ratio (95% CI) | Odds ratio (95% CI) | Odds ratio (95% CI) | Odds ratio (95% CI) |
| Continuous (per 1-SD increase) | 1.43 (1.17, 1.74) | 1.39 (1.07, 1.81) | 1.41 (1.08, 1.86) | 1.24 (0.86, 1.79) |
| SHR median |  |  |  |  |
| ≤0.81 | Reference | Reference | Reference | Reference |
| >0.81 | 5.38 (1.86, 15.58) | 3.64 (1.23, 10.82) | 3.58 (1.19, 10.78) | 3.29 (1.02, 10.61) |
| SHR tertiles |  |  |  |  |
| T1 (0.74<) | Reference | Reference | Reference | Reference |
| T2 (0.74-0.93) | 2.07 (0.38, 11.37) | 1.51 (0.26, 8.64) | 1.51 (0.26, 8.70) | 1.54 (0.24, 9.69) |
| T3 (>0.93) | 9.88 (2.31, 42.24) | 6.72 (1.52, 29.65) | 6.59 (1.47, 29.54) | 5.27 (1.12, 24.78) |
| P for trend | <0.001 | 0.002 | 0.002 | 0.013 |

Model 1: adjusted for age, sex, smoking status, alcohol consumption, heart rate, BMI, SBP, and DBP.

Model 2: adjusted for variables in Model 1+ WBC, RBC, HB, PLT, triglyceride, HDL-C, LDL-C, HbA1c, ALT, AST, GGT, albumin/globulin ratio, Scr, BUN, UA, potassium, and sodium.

Model 3: adjusted for variables in Model 2+ hypertension, coronary artery disease, diabetes mellitus, cancer, previous haemorrhagic stroke, and previous ischemic stroke.

Abbreviations: SD = standard deviation. Other abbreviations, see Table 1.

**Table S5**. Sensitivity analysis excludes participants with any missing values.

| Exposure | Crude model | Model 1 | Model 2 | Model 3 |
| --- | --- | --- | --- | --- |
|  | Odds ratio (95% CI) | Odds ratio (95% CI) | Odds ratio (95% CI) | Odds ratio (95% CI) |
| Continuous (per 1-SD increase) | 1.52 (1.31, 1.77) | 1.43 (1.21, 1.68) | 1.33 (1.11, 1.60) | 1.27 (1.05, 1.54) |
| SHR median |  |  |  |  |
| ≤0.81 | Reference | Reference | Reference | Reference |
| >0.81 | 3.70 (2.00, 6.84) | 3.26 (1.75, 6.08) | 2.93 (1.54, 5.58) | 2.63 (1.37, 5.04) |
| SHR tertiles |  |  |  |  |
| T1 (0.74<) | Reference | Reference | Reference | Reference |
| T2 (0.74-0.93) | 2.31 (0.95, 5.64) | 2.34 (0.95, 5.72) | 2.35 (0.95, 5.82) | 2.19 (0.88, 5.47) |
| T3 (>0.93) | 5.62 (2.51, 12.60) | 4.83 (2.14, 10.92) | 4.40 (1.90, 10.18) | 3.90 (1.67, 9.11) |
| P for trend | <0.001 | <0.001 | <0.001 | 0.001 |

Model 1: adjusted for age, sex, smoking status, alcohol consumption, heart rate, BMI, SBP, and DBP.

Model 2: adjusted for variables in Model 1+ WBC, RBC, HB, PLT, triglyceride, HDL-C, LDL-C, HbA1c, ALT, AST, GGT, albumin/globulin ratio, Scr, BUN, UA, potassium, and sodium.

Model 3: adjusted for variables in Model 2+ hypertension, coronary artery disease, diabetes mellitus, cancer, previous haemorrhagic stroke, and previous ischemic stroke.

Abbreviations: SD = standard deviation. Other abbreviations, see Table 1.

**Table S6**. E-values for the observed associations between SHR and in-hospital mortality in patients with acute stroke.

|  | Crude model | Model 1 | Model 2 | Model 3 |
| --- | --- | --- | --- | --- |
| Observed association* (per 1-SD increase) | 1.53 (1.32, 1.77) | 1.43 (1.23, 1.68) | 1.31 (1.10, 1.55) | 1.26 (1.05, 1.52) |
| E-value for point estimate | 2.43 | 2.21 | 1.95 | 1.83 |

Model 1: adjusted for age, sex, smoking status, alcohol consumption, heart rate, BMI, SBP, and DBP.

Model 2: adjusted for variables in Model 1+ WBC, RBC, HB, PLT, triglyceride, HDL-C, LDL-C, HbA1c, ALT, AST, GGT, albumin/globulin ratio, Scr, BUN, UA, potassium, and sodium.

Model 3: adjusted for variables in Model 2+ hypertension, coronary artery disease, diabetes mellitus, cancer, previous haemorrhagic stroke, and previous ischemic stroke.

Abbreviations: SD = standard deviation. Other abbreviations, see Table 1.

**Table S7.** Associations of SHR and length of hospital stay in patients with acute ischemic stroke.

| Exposure | Crude model | Model 1 | Model 2 | Model 3 |
| --- | --- | --- | --- | --- |
|  | β (95% CI) | β (95% CI) | β (95% CI) | β (95% CI) |
| Continuous (per 1-SD increase) | 0.88 (0.55, 1.21) | 0.84 (0.51, 1.18) | 0.80 (0.47, 1.14) | 0.75 (0.41, 1.09) |
| SHR median |  |  |  |  |
| ≤0.81 | Reference | Reference | Reference | Reference |
| >0.81 | 1.30 (0.64, 1.95) | 1.20 (0.54, 1.85) | 1.09 (0.43, 1.75) | 0.93 (0.27, 1.60) |
| SHR tertiles |  |  |  |  |
| T1 (0.74<) | Reference | Reference | Reference | Reference |
| T2 (0.74-0.93) | 0.35 (-0.45, 1.15) | 0.19 (-0.61, 0.99) | 0.20 (-0.60, 1.00) | 0.40 (-0.39, 1.19) |
| T3 (>0.93) | 1.50 (0.69, 2.30) | 1.34 (0.53, 2.16) | 1.24 (0.42, 2.05) | 0.99 (0.18, 1.81) |
| P for trend | <0.001 | 0.001 | 0.003 | 0.018 |

Model 1: adjusted for age, sex, smoking status, alcohol consumption, heart rate, BMI, SBP, and DBP.

Model 2: adjusted for variables in Model 1+ WBC, RBC, HB, PLT, triglyceride, HDL-C, LDL-C, HbA1c, ALT, AST, GGT, albumin/globulin ratio, Scr, BUN, UA, potassium, and sodium.

Model 3: adjusted for variables in Model 2+ hypertension, coronary artery disease, diabetes mellitus, cancer, previous haemorrhagic stroke, and previous ischemic stroke.

Abbreviations: SD = standard deviation. Other abbreviations, see Table 1.

**Table S8.** Associations of SHR and length of hospital stay in patients with acute haemorrhagic stroke.

| Exposure | Crude model | Model 1 | Model 2 | Model 3 |
| --- | --- | --- | --- | --- |
|  | β (95% CI) | β (95% CI) | β (95% CI) | β (95% CI) |
| Continuous (per 1-SD increase) | 0.73 (0.32, 1.13) | 0.57 (0.16, 0.98) | 0.51 (0.10, 0.92) | 0.64 (0.21, 1.07) |
| SHR median |  |  |  |  |
| ≤0.81 | Reference | Reference | Reference | Reference |
| >0.81 | 1.52 (0.66, 2.38) | 1.16 (0.30, 2.03) | 1.05 (0.19, 1.91) | 0.92 (0.03, 1.81) |
| SHR tertiles |  |  |  |  |
| T1 (0.74<) | Reference | Reference | Reference | Reference |
| T2 (0.74-0.93) | 0.22 (-0.84, 1.29) | 0.04 (-1.02, 1.10) | 0.31 (-0.75, 1.36) | 0.41 (-0.63, 1.45) |
| T3 (>0.93) | 1.11 (0.07, 2.15) | 0.70 (-0.36, 1.76) | 0.69 (-0.36, 1.74) | 0.46 (-0.63, 1.54) |
| P for trend | 0.036 | 0.197 | 0.198 | 0.401 |

Model 1: adjusted for age, sex, smoking status, alcohol consumption, heart rate, BMI, SBP, and DBP.

Model 2: adjusted for variables in Model 1+ WBC, RBC, HB, PLT, triglyceride, HDL-C, LDL-C, HbA1c, ALT, AST, GGT, albumin/globulin ratio, Scr, BUN, UA, potassium, and sodium.

Model 3: adjusted for variables in Model 2+ hypertension, coronary artery disease, diabetes mellitus, cancer, previous haemorrhagic stroke, and previous ischemic stroke.

Abbreviations: SD = standard deviation. Other abbreviations, see Table 1.

**Table S9**. Sensitivity analysis excludes participants with any missing values.

| Exposure | Crude model | Model 1 | Model 2 | Model 3 |
| --- | --- | --- | --- | --- |
|  | β (95% CI) | β (95% CI) | β (95% CI) | β (95% CI) |
| Continuous (per 1-SD increase) | 0.90 (0.60, 1.21) | 0.84 (0.54, 1.15) | 0.80 (0.49, 1.11) | 0.75 (0.43, 1.06) |
| SHR median |  |  |  |  |
| ≤0.81 | Reference | Reference | Reference | Reference |
| >0.81 | 1.38 (0.76, 2.01) | 1.23 (0.60, 1.86) | 1.11 (0.49, 1.74) | 0.89 (0.26, 1.53) |
| SHR tertiles |  |  |  |  |
| T1 (0.74<) | Reference | Reference | Reference | Reference |
| T2 (0.74-0.93) | 0.31 (-0.46, 1.08) | 0.11 (-0.66, 0.88) | 0.18 (-0.59, 0.95) | 0.28 (-0.47, 1.04) |
| T3 (>0.93) | 1.50 (0.74, 2.26) | 1.28 (0.52, 2.05) | 1.19 (0.42, 1.95) | 0.86 (0.08, 1.63) |
| P for trend | <0.001 | 0.001 | 0.002 | 0.031 |

Model 1: adjusted for age, sex, smoking status, alcohol consumption, heart rate, BMI, SBP, and DBP.

Model 2: adjusted for variables in Model 1+ WBC, RBC, HB, PLT, triglyceride, HDL-C, LDL-C, HbA1c, ALT, AST, GGT, albumin/globulin ratio, Scr, BUN, UA, potassium, and sodium.

Model 3: adjusted for variables in Model 2+ hypertension, coronary artery disease, diabetes mellitus, cancer, previous haemorrhagic stroke, and previous ischemic stroke.

Abbreviations: SD = standard deviation. Other abbreviations, see Table 1.

**Table S10**. E-values for the observed associations between SHR and length of hospital stay in patients with acute stroke.

|  | Crude model | Model 1 | Model 2 | Model 3 |
| --- | --- | --- | --- | --- |
| Observed association* (per 1-SD increase) | 0.83 (0.55, 1.12) | 0.77 (0.48, 1.06) | 0.72 (0.43, 1.01) | 0.70 (0.40, 1.00) |
| E-value for point estimate | 4.01 | 4.04 | 4.06 | 4.07 |

Model 1: adjusted for age, sex, smoking status, alcohol consumption, heart rate, BMI, SBP, and DBP.

Model 2: adjusted for variables in Model 1+ WBC, RBC, HB, PLT, triglyceride, HDL-C, LDL-C, HbA1c, ALT, AST, GGT, albumin/globulin ratio, Scr, BUN, UA, potassium, and sodium.

Model 3: adjusted for variables in Model 2+ hypertension, coronary artery disease, diabetes mellitus, cancer, previous haemorrhagic stroke, and previous ischemic stroke.

Abbreviations: SD = standard deviation. Other abbreviations, see Table 1.

## 2.2 Supplementary Figures

**
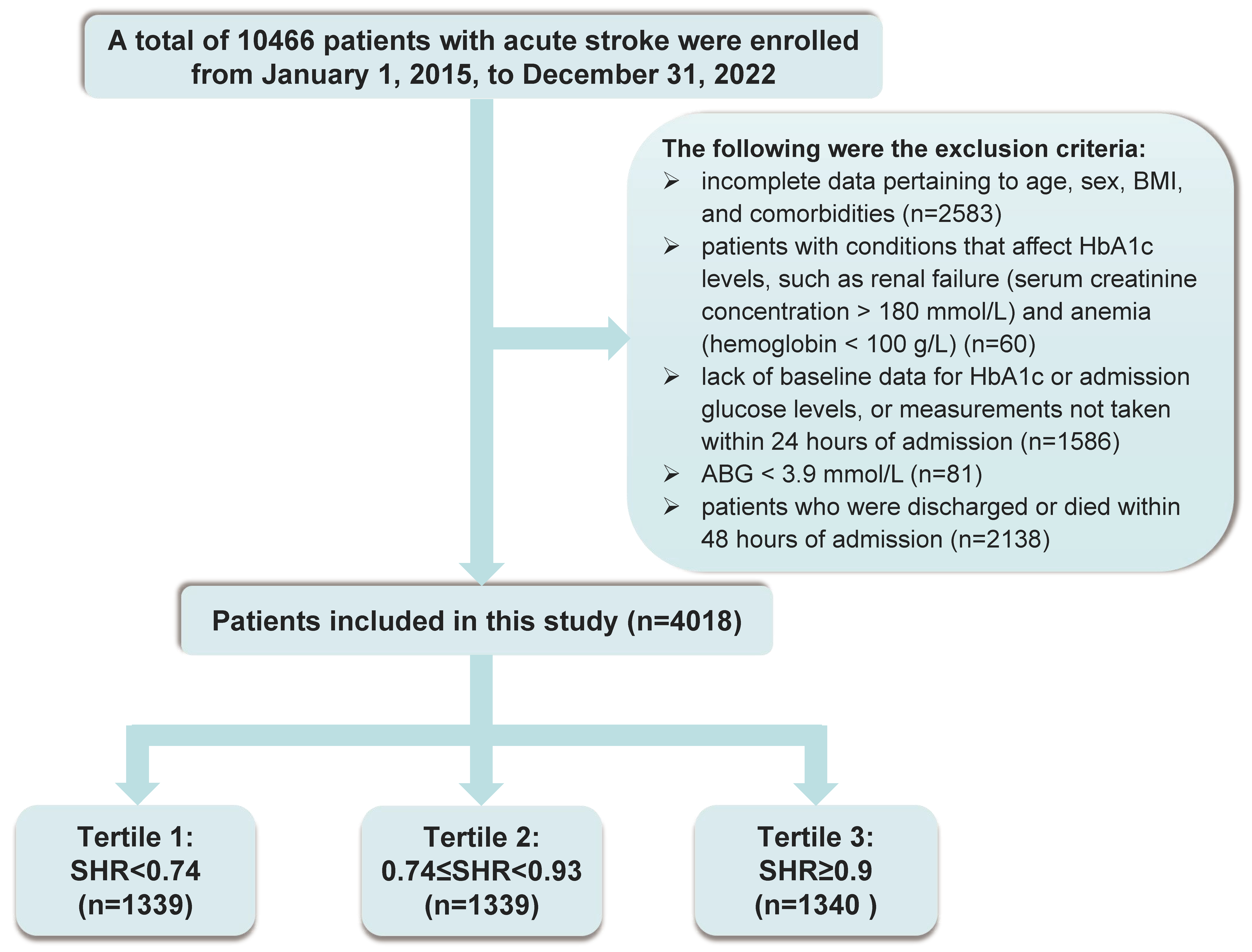
**

**Figure S1** Flowchart of the study.


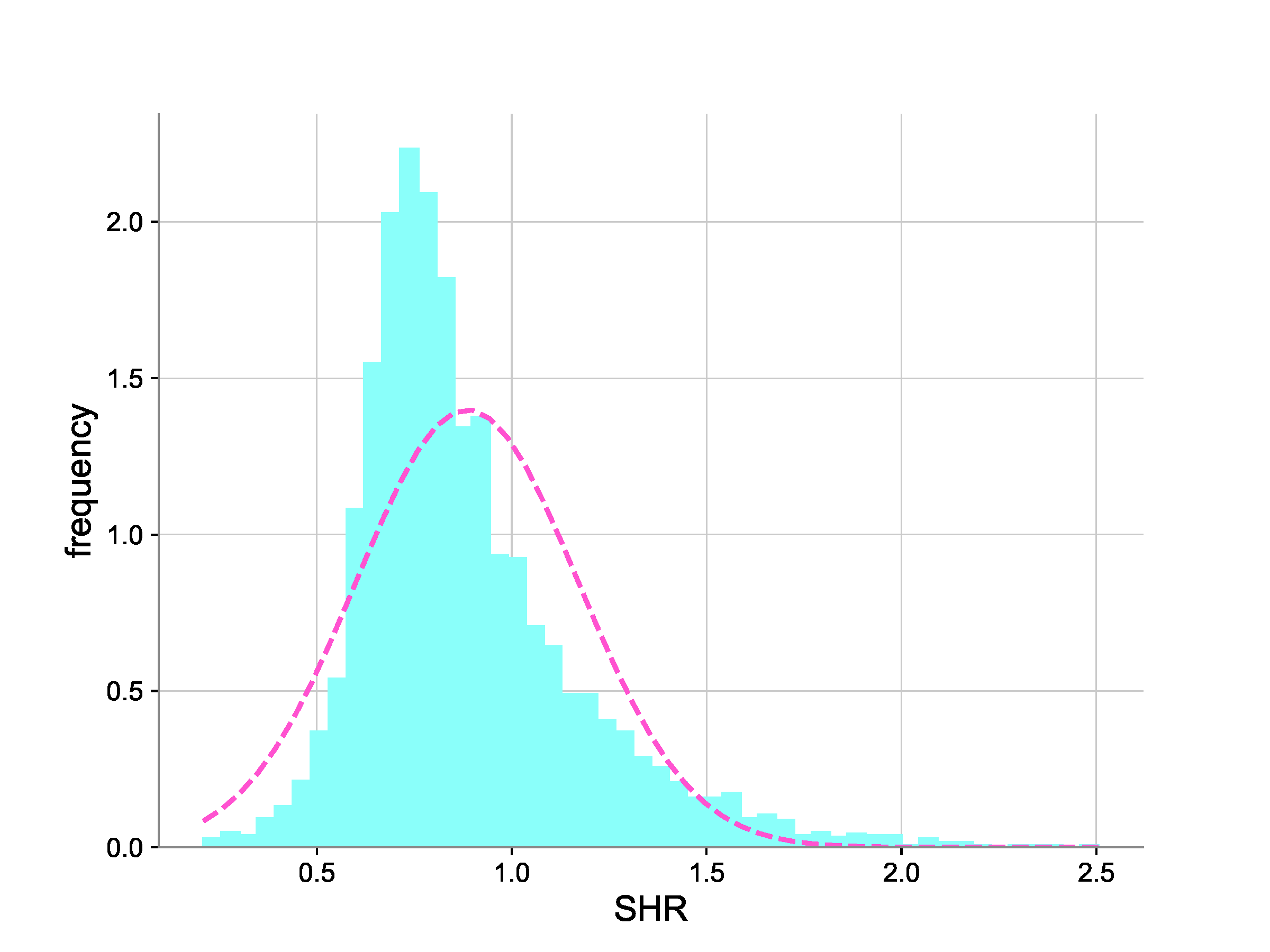


**Figure S2** Frequency distribution of SHR of study participants.


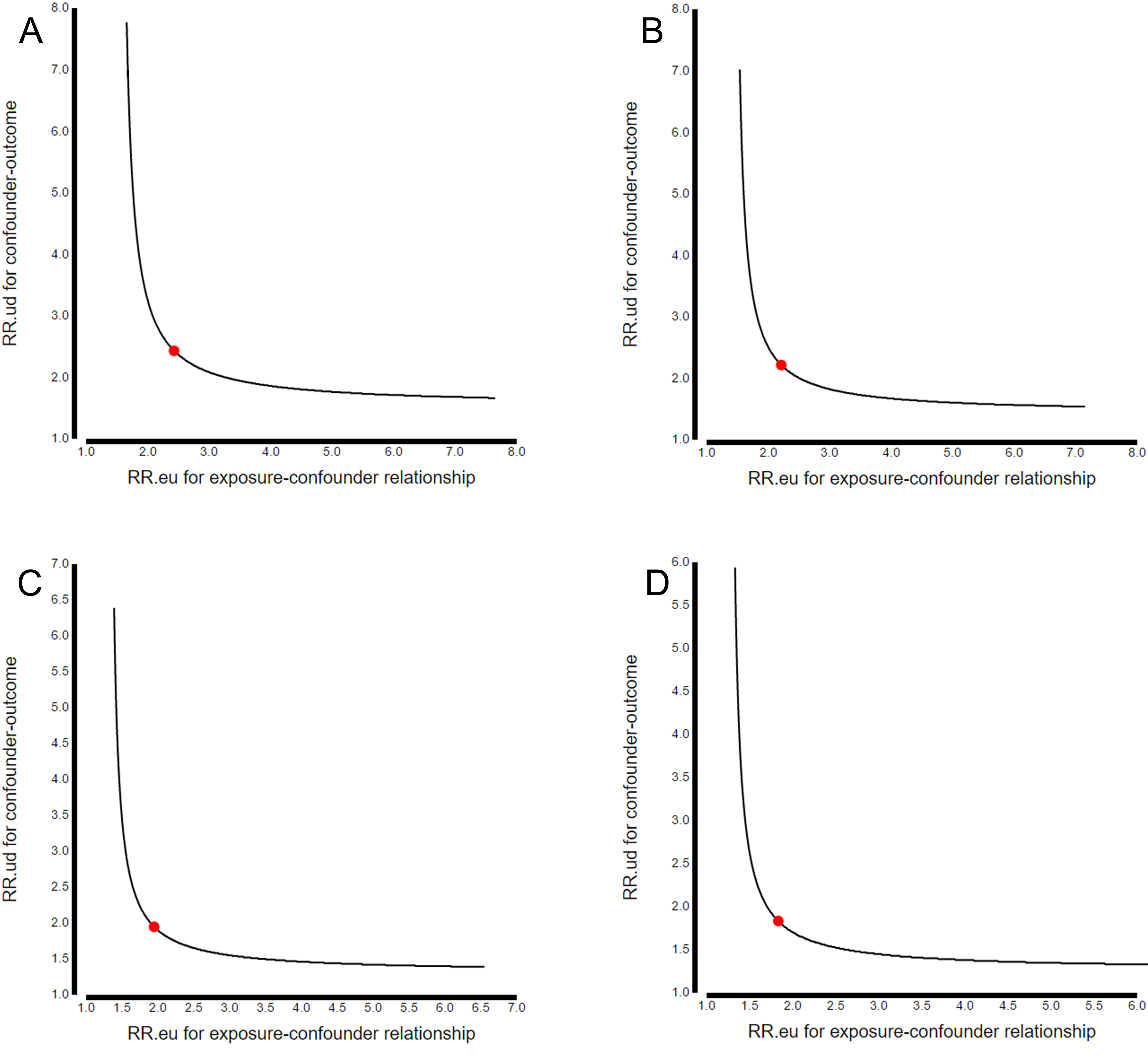


**Figure S3** E-values for multivariable logistic regression models. (A) crude model, (B) model 1, (C) model 2, and (D) model 3.

Model 1: adjusted for age, sex, smoking status, alcohol consumption, heart rate, BMI, SBP, and DBP.

Model 2: adjusted for variables in Model 1+ WBC, RBC, HB, PLT, triglyceride, HDL-C, LDL-C, HbA1c, ALT, AST, GGT, albumin/globulin ratio, Scr, BUN, UA, potassium, and sodium.

Model 3: adjusted for variables in Model 2+ hypertension, coronary artery disease, diabetes mellitus, cancer, previous haemorrhagic stroke, and previous ischemic stroke.


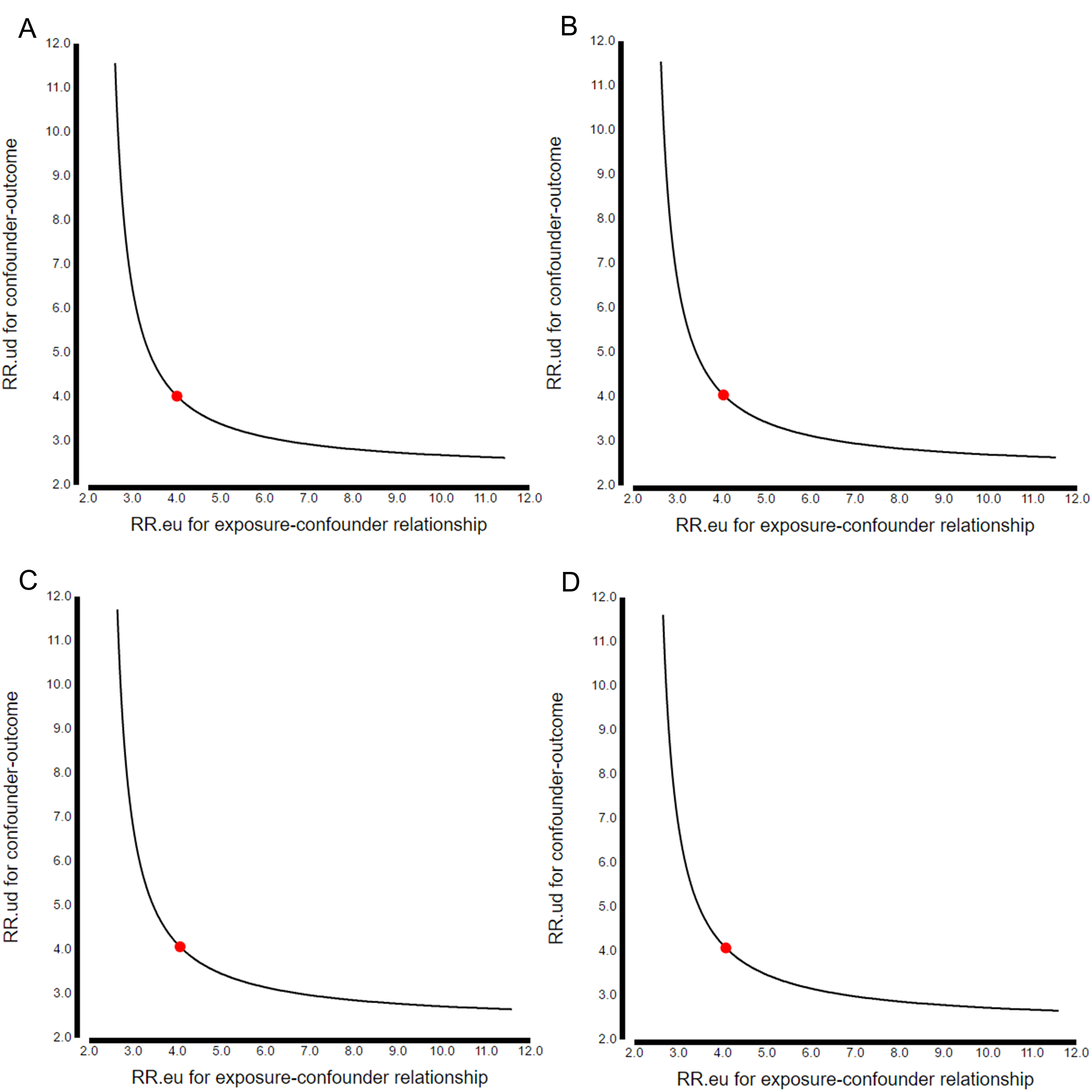


**Figure S4** E-values for multivariable linear regression models. (A) crude model, (B) model 1, (C) model 2, and (D) model 3.

Model 1: adjusted for age, sex, smoking status, alcohol consumption, heart rate, BMI, SBP, and DBP.

Model 2: adjusted for variables in Model 1+ WBC, RBC, HB, PLT, triglyceride, HDL-C, LDL-C, HbA1c, ALT, AST, GGT, albumin/globulin ratio, Scr, BUN, UA, potassium, and sodium.

Model 3: adjusted for variables in Model 2+ hypertension, coronary artery disease, diabetes mellitus, cancer, previous haemorrhagic stroke, and previous ischemic stroke.

**References**

[1] VanderWeele TJ, Ding P. Sensitivity Analysis in Observational Research: Introducing the E-Value. Ann Intern Med. 2017. 167(4): 268-274.
